# Perception, prestige and PageRank

**DOI:** 10.1371/journal.pone.0216783

**Published:** 2019-05-28

**Authors:** David Zeitlyn, Daniel W. Hook

**Affiliations:** 1 Institute of Social and Cultural Anthropology, School of Anthropology and Museum Ethnography, University of Oxford, Oxford, United Kingdom; 2 Digital Science, London, United Kingdom; 3 Centre for Complexity Science, Imperial College London, London, United Kingdom; 4 Department of Physics, Washington University in St Louis, St Louis, MO, United States of America; Public Library of Science, UNITED KINGDOM

## Abstract

Academic prestige is difficult to quantify in objective terms. Network theory offers the opportunity to use a mathematical formalism to model both the prestige associated with an academic and the relationships between academic colleagues. Early attempts using this line of reasoning have focused on intellectual genealogy as constituted by supervisor student networks. The process of examination is critical in many areas of study but has not played a part in existing models. A network theoretical “social” model is proposed as a tool to explore and understand the dynamics of prestige in the academic hierarchy. It is observed that such a model naturally gives rise to the idea that the prestige associated with a node in the graph (the prestige of an individual academic) can be viewed as a dynamic quantity that evolves with time based on both local and non-local changes in the properties in the network. The toy model studied here includes both supervisor-student and examiner-student relationships. This gives an insight into some of the key features of academic genealogies and naturally leads to a proposed model for “prestige propagation” on academic networks. This propagation is not solely directed forward in time (from teacher to progeny) but sometimes also flows in the other direction. As collaborators do well, this reflects well on those with whom they choose to collaborate and those that taught them. Furthermore, prestige as a quantity continues to be dynamic even after the end of a relationship or career. Given that time ordering of relationships on the network are implicit but that measures such as betweenness are independent of this implicit time dependence: the success of a PhD student later in their career can improve the prestige of their doctoral supervisor. Thus, prestige can be interpreted to have dynamics that flow both forward and backward in time.

## Introduction

The use of network theory to represent and study the nature of academic relationships is not new. Co-authorship and citation graphs are used, to meet the data requirements of academic appointments panels, tenure review committees, funder processes and government assessment activities around the world [1]. However, outside the context of research evaluation, there remain some practical and important uses of graph theory: To understand the semantic proximity of research fields; to observe and predict the emergence of new fields; to quantify the connectedness of individual researchers; and, to locate researchers who form pivotal relationships and bridge different fields with their research.

The idea of an intellectual genealogy as a set of relationships between supervisors and research students is well established and leads naturally to the concept of an intellectual lineage [2,3]. While the supervisor-supervisee relationship is the most commonly recorded and studied relationship, it is not the only link that can be used to formulate an intellectual genealogy. Based on an empirical case study (used as a toy model), this paper proposes a framework to quantify prestige ranking on an academic network. Researchers habitually try to contextualise colleagues by their relationships. It is generally accepted that in the early stages of a career whether someone’s supervisor was “good”, whether their examiner was “well-known” and whether their research collaborators are “recognised” are important factors in later success. Of course, “good”, “well known” and “recognised” are all subjectively defined but it is clear that the perception generated by being “chosen” by accepted prestigious individuals is an important one, not just in academia. Such observations have recently become a “hot topic” as analysis by Ma et al. [4] suggests that women benefit less from this ecosystem than their male counterparts. We conjecture that a deeper analysis of the network approach that we have suggested here would highlight further examples of women who have been overlooked but who have had significant and unsung influence on a field.

Over the last 50 years’ doctorates have become established as a gateway to a career in the academy. As a result, doctorates have become a common currency in academia and are now often a rapid proxy to help to contextualise a researcher in some mental map of the research landscape. That is to say that especially at the beginning of an academic career, before an academic has had time to acquire awards, write papers, gain grants or garner citations, commonly asked questions include: Where did the researcher in question study? In what department? And, With whom? The answers to these questions instantly give a picture of the researcher in the internal ranking system of the questioner. If the institution is well known, the department of note or the supervisor famous, then an expectation is already set by this context. This impression is not easy to make tangible since it is a highly-subjective measure of academic prowess and is personal to each evaluator and their own academic collaborations, interactions and experiences. Hence, while many decisions are influenced by this type of prestige measure, it is not easy to define and is impossible defend in objective terms.

The emergence of network theory as tool to model and encode social phenomena using mathematical formalism mean that there is a natural means of capturing both the relationships described above and codifying not only their explicit importance (direct prestige from winning some kind of esteemful award) as well as their inferred significance (indirect prestige derived from being located in the proximate network of one or more award winners). This formalism is powerful as it allows us to derive network properties associated with academics in the graph including their connectedness, betweenness or simply their rank. While ranking is distasteful to most academics, ranking schemes in network theory allow us to uncover less obvious results since there is not a direct relationship between the winner of the most awards and the top position in a ranking. Ranking in a Google PageRank sense gives us an understanding of the most influential (or popular) nodes in a graph. Popularity in the current context can be thought of as a proxy for overall proximity to prestige.

What do we mean by “PageRanking” academics? There are three critical facets that make this approach interesting in our context. Firstly, PageRank gives us a quantitative approach that allows us to define a metric that gives us a natural ordering for a group of academics, so long as they participate in a connected network. Secondly, PageRank does not correspond to a trivial ordering of those with the highest number of awards being top of the ranking but is a more subtle measure that takes into account position in the network–proximity to highly-ranked participants increases rank even if no prize or award has been won by a specific participant. Finally, PageRank is not a pairwise comparison of each two participant but rather is a way in which local and global properties of a network can be brought in contact to inform a ranking.

A further advantage of a network-based approach is that awards and prizes are relatively rare in academia and awards are often attracted to individuals who have previously won awards. As a result, the distribution of awards is very inhomogeneous in the community and also on an academic genealogy. Connects in the network are also scarce. This means that any non-network ranking is automatically unfair since it prejudices results either to small sub-communities or to those with large numbers of awards and those who are not luckily enough to win awards directly are overlooked and become invisible. A similar problem was accessed by Keener et al. [5] in their study of ranking college football teams where league-based approaches did not necessarily mean that the best teams won the competition since not every team was able to play every other team due to time and geographical constraints. This resulted in a sparse set of connections where information was not optimally leveraged to locate the best team.

In this paper, we carry out an initial exploration of a dataset that we have created by bringing together data on one subject area from several sources and formulate some conjectures based on these data and an initial analysis. In particular, we look beyond the supervisor-supervisee doctoral thesis graph. We consider connections mediated by the doctoral thesis but instead of considering only the supervisory relationship we also consider the graph formed by taking the examiners (so covering all parents) of a doctoral thesis and the candidate (child).

There is a rich and complex sociology behind thesis examination that needs to be understood before we can extend the supervisor-supervisee network model with confidence. Our initial approach was to consider all dissertations (masters level, and doctoral level) and weight the relationships associated with the level of the qualification, but this adds significant additional complexity: It is unclear what would constitute a fair weighting, it is unclear whether the a masters dissertation included novel research or was simply a literature review and, finally, the number of masters students who do not carry on to academia is high and hence many of these nodes remain unconnected and merely add data complexity without adding content. Additionally, in many universities, a student “failing” a doctoral qualification may be given an MPhil or other Master-level qualification. It is not easy to tell from the records that are kept publicly which course was originally intended. Having a number of failed students may be an interesting signal to study but could be symptomatic of many different factors and hence becomes quickly too complex (not to mention controversial) to access as a problem in an initial study. As a result of these complicating factors, we have focussed on successful doctoral theses. This simplifies some of the data issues and focuses the study on a richer network where the participants are more likely to remain part of the research ecosystem and hence contribute to the graph more actively by becoming examiners themselves. Another weighting effect that should be considered is the relative importance of examining versus supervising from field to field. Although the supervisor spends more time with the student during the performance of their work, in some fields, the examiner of the thesis will be rated at a similar level of importance (especially where there are doctoral committees). Since we are dealing with modelling perception in this case, the relative importance between supervisor and examiner may even be examiner (and supervisor) dependent. In the case that an extremely eminent individual examines the thesis, this may have more bearing than a relatively less known supervisor.

Further complexities still exist even with our initial simplification: In some universities, defences are public and there is a team of examiners, in other institutions, the viva is closed and attendance is limited to the candidate and two examiners. In some areas it is acceptable for a candidate’s supervisor to attend the viva, in others the supervisor may be one of the examiners, in yet others the supervisor may not be allowed to attend. Some universities require that one or more of the examiners must be from outside the institution. Many institutions require that there is no formal relationship between the candidate and the examiner (i.e. they may not have written a paper together prior to the examination).

In some fields the external examiner is viewed as being almost as important as the supervisor since they act as the gatekeeper who allows a candidate to pass from student to a member of the academy. Passing a defence with a well-known, respected or highly-established academic can be a mark of prestige and can be helpful for a young researcher. Of course, choosing a very well-known academic as a panellist can be risky since failing a viva can be quickly reported around the academic social network and can reflect badly on the student, supervisor, and even the department or university. Often, panellists are co-authors of one of the supervisors. All these factors contribute to a subtle and interesting network for study.

There are also other important effects to bear in mind when considering the network of panellists that relate to institutional prestige and to practicality: It may be that there is a systemic internal bias for institutions to choose examiners from universities that are currently deemed to have higher status than the source institution; for practical purposes geography can play a significant role in that it is easier and cheaper to get an external examiner from a nearby university than from further afield where logistical issues makes the arrangement more difficult or more expensive. Of course, the factors of status and geography interfere in a complex fashion. Furthermore, the choice of possible externals is also influenced by judgements by supervisors about the merits of individual students. A chosen examiner must be available, must know the topic concerned and be appropriate both in terms of prestige and status. It is plausible that across the academy the effects of these merit judgements cancel out leaving distance and status as the most significant factors.

A number of data sets suggest themselves as a starting point for studying this area: there are several online academic genealogy projects holding PhD student-supervisor data [6,7]: We collated data from the following online collaborative sources: Academic tree http://academictree.org/; Primatology Tree http://www.physanthphylogeny.org/ and PhD Tree http://phdtree.org/. Goydar worked on the comparative prestige of sociology departments based not on publications but on the prestige of the universities awarding the PhDs of staff members [8]. The latter approach seems to capture the entrenched position of Oxford, Cambridge and few other Russell Group Universities in the UK together with the Ivy League and similar high-profile institutions in the US. It is interesting to note that while the identity of an institution typically won’t change in the short term, its level of prestige or prestige may, although typically within the lifetime of a single academic career the prestige of an institution can mostly be considered to be stable. There may be more, faster variation in the prestige of individual departments [9].

This paper is organised as follows: In Sec. 1 we explain how we created and curated the case study dataset that we then go on to use as a toy model to explore the ideas laid out above. In Sec. 2 we summarise the analysis of the dataset. In Sec. 3 we discuss a potential model to explore prestige propagation across an academic network. In Sec. 4 we include a brief discussion of the research and suggest some directions for further research.

### 1. Construction of the primatology dataset

For our study, we have created a dataset describing academic relationships in the field of primatology. We describe the creation of the dataset in detail below. From the genealogical or kinship approach that is our focus in this paper, each node in our network corresponds to a person. Each examination defines a relationship between a set of nodes or individuals. The role that each person plays in each exchange is not a property of the node but rather of the link (the degree/examination). Hence, there are two types of directed edge in our network. Each edge corresponds to a single instance of a degree / examination and hence is associated with a single student who is undergoing an examination. There are two types of edge: supervisor-student edges and examiner-student edges and so a single examination in codified in a number of different edges. We have not attempted to codify other types of relationship in the graph such as examiner-examiner, supervisor-examiner or supervisor-supervisor edges–such relationships are left to be inferred from the graph through the examination events.

**Fig 1:**
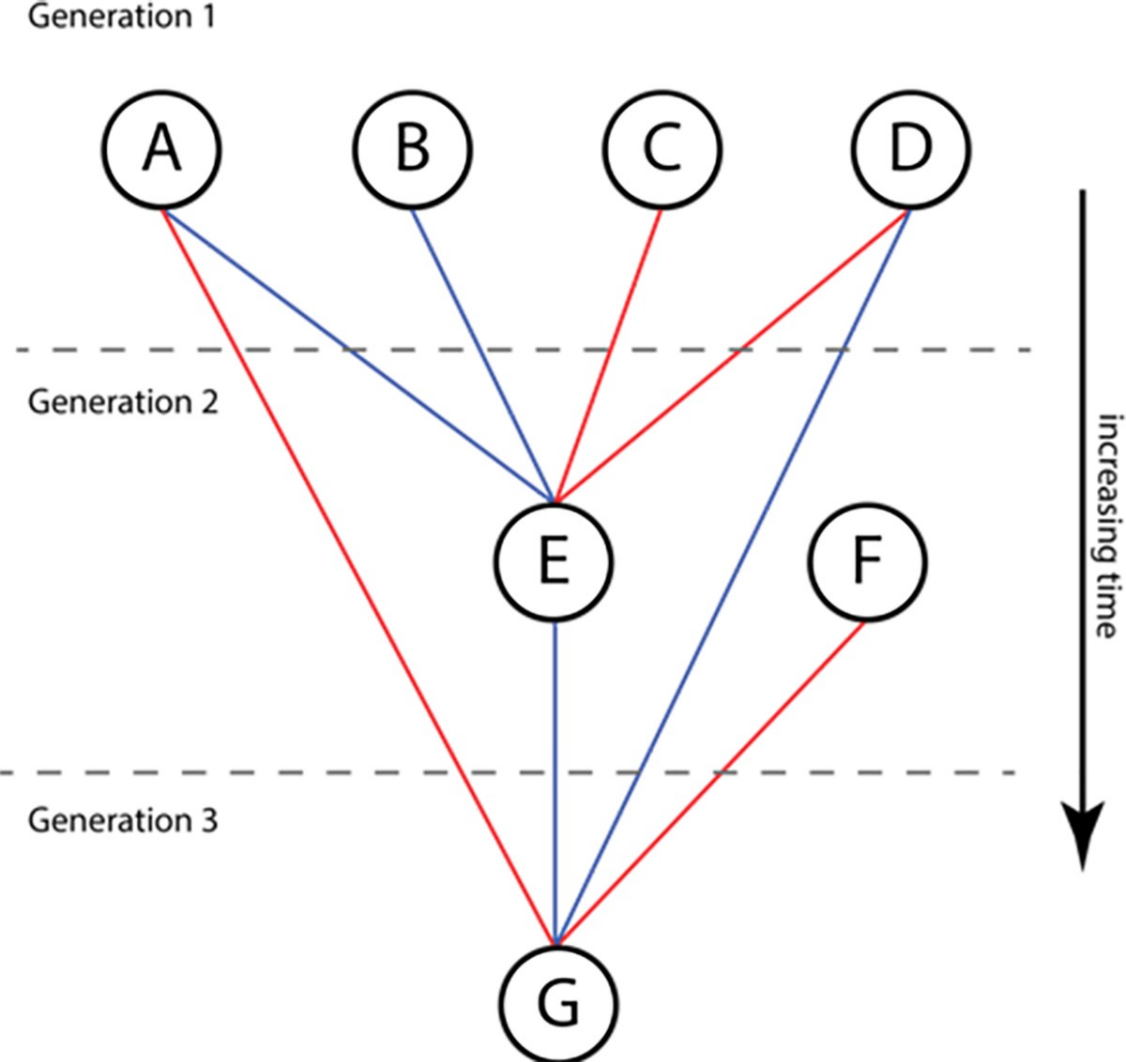
Simple model for academic hierarchy involving both supervisor-student links and examiner-examinee links. Red lines represent examination relationships and blue lines represent supervisor/advisor relationships. In this diagram, A and B supervise E, who is examined by C and D; E then supervises G with D, who is in turn examined by A and F.

There is an implicit hierarchy in the network since there are dates associated with the examination events and the assumption (in general) is that the least experienced researcher will be the one undergoing examination or supervision. As a result, this is a temporal (edge) network graph of the type discussed by Holme & Saramaki [10] or Masuda and Lambiotte [11], although we have not exploited this in our work to date. Fig 1 shows a simple instance of a 3-generation academic genealogy in which the student E in Generation 2 is the supervisor of G in the 3^rd^ generation and who draws on her previous supervisors as examiners and is co-supervising the student with one of her examiners.

In practical terms, finding data to formulate this graph is challenging and many value judgements must be made to create something that can sensibly represent an area. As we were looking for a graph containing links based on examinations, we needed to find a field where data was sufficiently readily available and the intellectual genealogy work of Sussman and Kelley was generously shared in order to provide a starting point for our own research [12]. The database of PhD supervisor-supervisee relationships provided by Sussman and Kelley was heavily supplemented by adding in doctoral examiner and committee data for the first time.

Beyond data availability, there are several reasons that we chose primatology for our toy model. Primatology is an interdisciplinary subject spanning observational field studies via captive studies to genetics. Primatologists are to be found in Zoology, Psychology, Biology, Medicine, Archaeology and Anthropology departments. This means that we are able to access a number of different subject styles to get a more universal picture. From a practical perspective, the size of the field of primatology is sufficient to be interesting but small enough that we are able to handle the data for cleaning purposes as well as for the purposes of drawing meaningful conclusions.

Kelley and Sussman’s original dataset concentrated on American field primatologists and identifies the supervisory linage of Sherwood Washburn as the defining characteristic of the discipline. In the current work, we cast our net wider restricting ourselves to neither a methodological orientation or a sub-continent. Nevertheless, Washburn’s lineage emerges as central. From a technical perspective, we have set further limits on what constitutes primatology for our purposes–specifically, we have not included theses on humans. Decisions on the inclusion of theses in the dataset were made by Zeitlyn: If a title was ambiguous in whether the focus was on human primates the abstract of the thesis was examined to clarify scope. The thesis was included in the dataset only if the thesis did not consider human primates as its principal area of interest. It was not possible to automate or map this process to an algorithm. Classification of work in a narrow field remains a challenging problem. In order to do this in a reproducible manner using machine learning or classification algorithms, it is necessary to possess a sufficiently large body of pre-tagged data, this is something this is not readily available in the area of primatology (in fact the current dataset associated with this paper may be the first to provide a learning set on which to base algorithmic approaches). We also need to specify what types of thesis are appropriate to include in our study: The theses that we deemed relevant for analysis had to meet the following two criteria: Firstly, only doctoral theses that resulted in an awarded qualification were included (this means that masters-level dissertations, even if related to research results were not considered); secondly, only theses related to non-human primatology were included. The core dataset was derived by searching the Proquest Dissertations & Theses database using the search string:

*subject(*"*Physical anthropology*"*) and (angwantibo or aye-aye or bonobo or bushbaby or galago or guenon or guereza or malbrouck or mandrill or muriqui or nycticebus or potto or talapoin or vervet) not (sifaka or loris or lemur or bushbaby or tarsier or marmoset or tamarin or capuchin or titi or saki or uakari or howler or macaque or mangabey or baboon or mangabey or drill or colobus or lutung or surili or douc or monkey or langur or gibbon or ape or orangutan or Gorilla or chimpanzee) or primatology)*

We include a number of exemplar cases here to illustrate the types of decision that have been made: Maki’s 2013 ‘The Biomechanics Of Spear Throwing: an Analysis of the Effects of Anatomical Variation on Throwing Performance, with Implications for the Fossil Record’ was deemed out of scope.

When considering the inclusion of supervisors and examiners we have taken the view that anyone supervising or examining a primatologist should be included (whatever their formal subject affiliation) since their supervision/examining has a bearing on primatology. Conversely, not all those supervised or examined by a primatologist may be primatologists so not all the students of a given parent (even of Washburn) have been included. This is a subject based project. The dataset that we compiled and studied is available, together with an interactive visualisation [13].

It is important to note that not all individuals considered to be primatologists are included in our network for the simple reason that their doctoral studies were not on primatology. Hence the doctorates of several prominent individuals who became primatologists only after completing a doctorate on other subjects are not included. Examples include Clarence Raymond Carpenter since his 1932 Stanford Ph.D. was in psychology and ornithology even though Kelley and Sussman describe him as ‘the first true field primatologist’ on the basis of his post-doctoral work. He is, however, included in our database as supervisor and examiner. Another similar case is that of Thelma Rowell.

Among other examples we note Dr Klára Petrželková, whose 2003 PhD from Masaryk University in the Czech Republic was on bats and so deemed out of scope. It was only subsequently that she started working on primates. Petrželková does, however, appear as a supervisor and examiner of primatologists. Other notable examples include James R. Roney, ‘Psychological and hormonal responses of men to sensory cues from women’ (2002) https://catalog.lib.uchicago.edu/vufind/Record/4733089; Harry Israel’s Stanford PhD on albino rats (1930). The last has a further significance since the author changed his name and subsequently was better known as Harry F. Harlow. Another, more recent case of male name changes is Adam Clark Arcadi who writes on his web page http://anthropology.cornell.edu/people/faculty-list/adam-clark-arcadi.cfm ‘Prior to 1996 I published under the name Adam P. Clark’, last accessed 1 Oct 2015. We have several cases of women changing name on marriage and / or divorce. This leads to challenges in carrying out person disambiguation on the dataset. While analysis has been carried out to use standard pattern matching approaches to deduplicate individuals in the data as well as some manual work, only in cases where we explicitly know about changes of name have we been able to take this into account in the underlying data.

Since in some systems of doctoral assessment supervisors are also examiners there was some duplication in the data. We left this data in the main dataset set since it may be of interest (it represents a meeting between the groups of co-examiners, the juries), but in the network analysis reported here we omitted examiners who were also supervisors, on the basis that their role in the examination process was different from the other examiners. They were present as a function of their role as supervisor, they had not been asked solely to assess the submitted thesis. We note this points to an ambiguity in some US doctoral committees, which are constituted from the start of the research and so combine supervisory and examining roles. We have taken the one or two committee members designated as ‘primary supervisors’ as such and treated the remainder of the committee as examiners.

In our data the maximum number of examinations for an individual is 56 and the maximum number of supervisions is 32 (not the same person). Furthermore, we note that some individuals have several doctorates (quite apart from honorary doctorates which we are not discussing at present). An extreme case is William Clement McGrew who has 3 doctorates at least 2 of which fit our criteria.

### 2. Key aspects of the dataset

As active primatologists Kelley and Sussman had a natural intuition that Washburn was a central figure in primatology and, indeed, were able to demonstrate the importance of his lineage in America. They also document some other lineages in other countries (e.g. that associated with Robert Hinde in Cambridge) and some others in America. In our work, we seek to remove the intuition component and show that network properties and statistics confirm intuition and can be used in place of intuition to identify key actors in a field.

In our analysis we used Pajek and the SNA package in R to calculate network statistics. Overall, we captured and codified 7557 examinations, 4628 supervisions related to a total of 8443 qualification “events”, comprising 9828 connections between examiners and vivas and, 3486 connections between supervisors and students. Table 1 summaries some of the key network statistics.

**Table 1:** Summary of key network statistics for primatology toy model including, number of disconnected components, size of largest connected component and network density.

Network	Number of nodes	Number of edges	Number of connected components	components containing more than 10 nodes	Nodes in largest comp	Percentage of network in largest comp	Network density
Examinations	7557	9828	486	31	4939	65.36527	0.0001721626
Supervisions	4628	3487	1188	27	1119	24.18414	0.0001628629
All	8443	13314	534	32	5764	68.27766	0.0001868398

**Fig 2:**
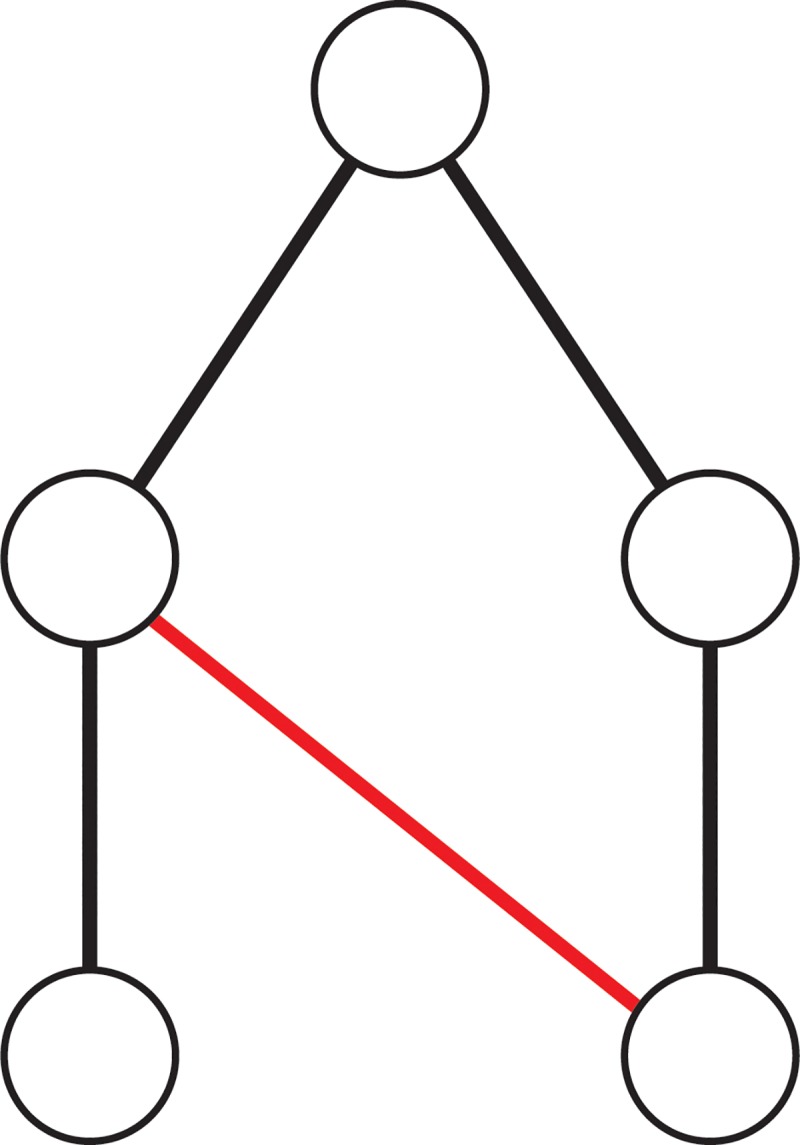
Simple closed loop showing the relationship where one examiner had the same intellectual "father" or "mother" as the supervisor of the student being examined. Black lines show supervisory relationships. Red lines show examiner relationships.

**Fig 3:**
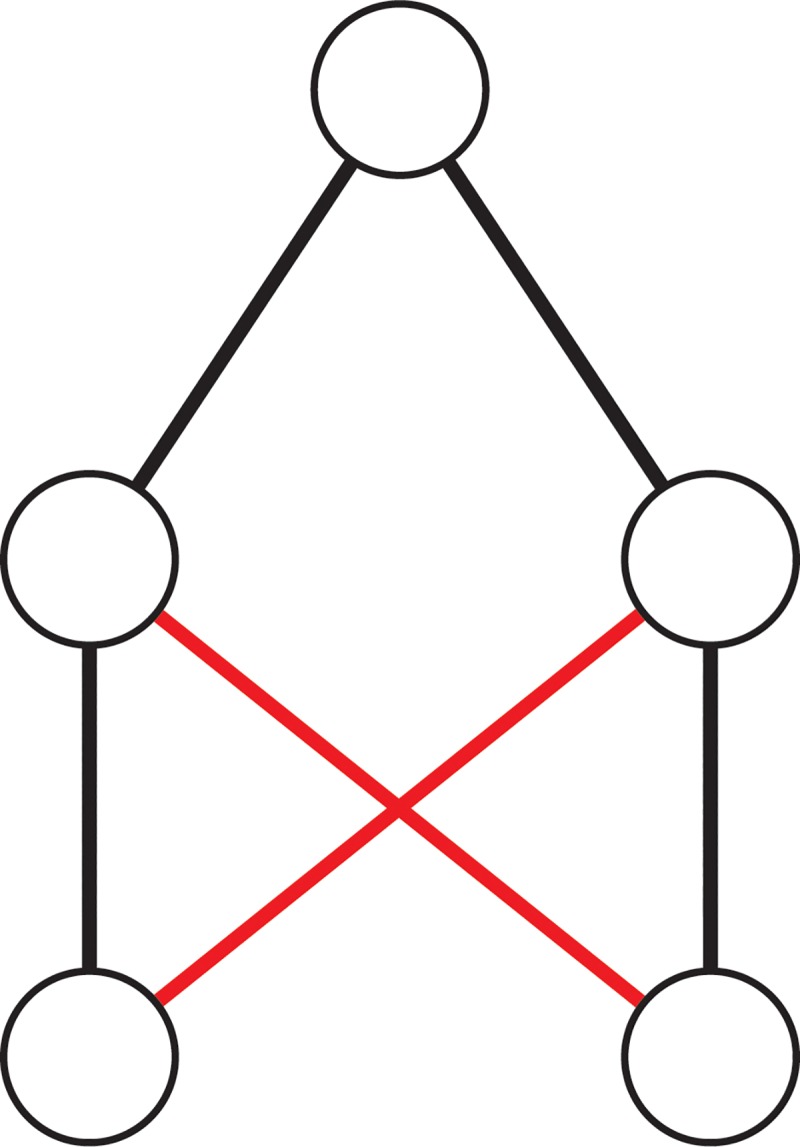
Incestuous relationship loop, in which two supervisors who are children of the same intellectual parent, examine each other's students. Black lines denote supervisory relationships. Red lines denote examiner relationships.

As an aside, we note that from the genealogical perspective, this dataset is fascinating as it allows us to transfer fundamental concepts from kinship theory such as “cousin marriage” and asymmetrical marriage among distant kin to explore their commonality in an academic setting. From a network perspective, these concepts are equivalent to asking whether the network contains closed loops or cycles (see Fig 2, Fig 3). Since our data are time-ordered, it is simple to remove closed loops if needed. The study of kinship concerns both the tracing of descent (the ways in which people identify themselves to one another) and the arrangement of marriage, who is marriageable and who cannot be married. Kinship Theory has been one of the central parts of academic anthropology for more than a century [14,15] and supplies an interesting set of comparator networks that motivate some questions about our current dataset from a non-traditional network-theoretic perspective.

Loops occur for a number of reasons: For example, hypothetically two students of the same supervisor might in time come to have their own students. When looking for an examiner, Supervisor A may think of their former peer as being suitable since they come from the same disciplinary tradition. This would create a very small cycle, see Fig 2.

We note that it might seem wrong to “return the favour” as this may be termed ‘incestuous’ see Fig 3. The tendency of universities to appoint their own graduands can also be loosely classified as an example of incestuous behaviour, but kinship theory suggests that this should more accurately described as a form of intellectual endogamy (see Godechot [16–21] whose title talks of ‘inbreeding’). Indeed, in subjects where the availability of experts is limited, certain endogamous behaviours are unavoidable.

**Fig 4:**
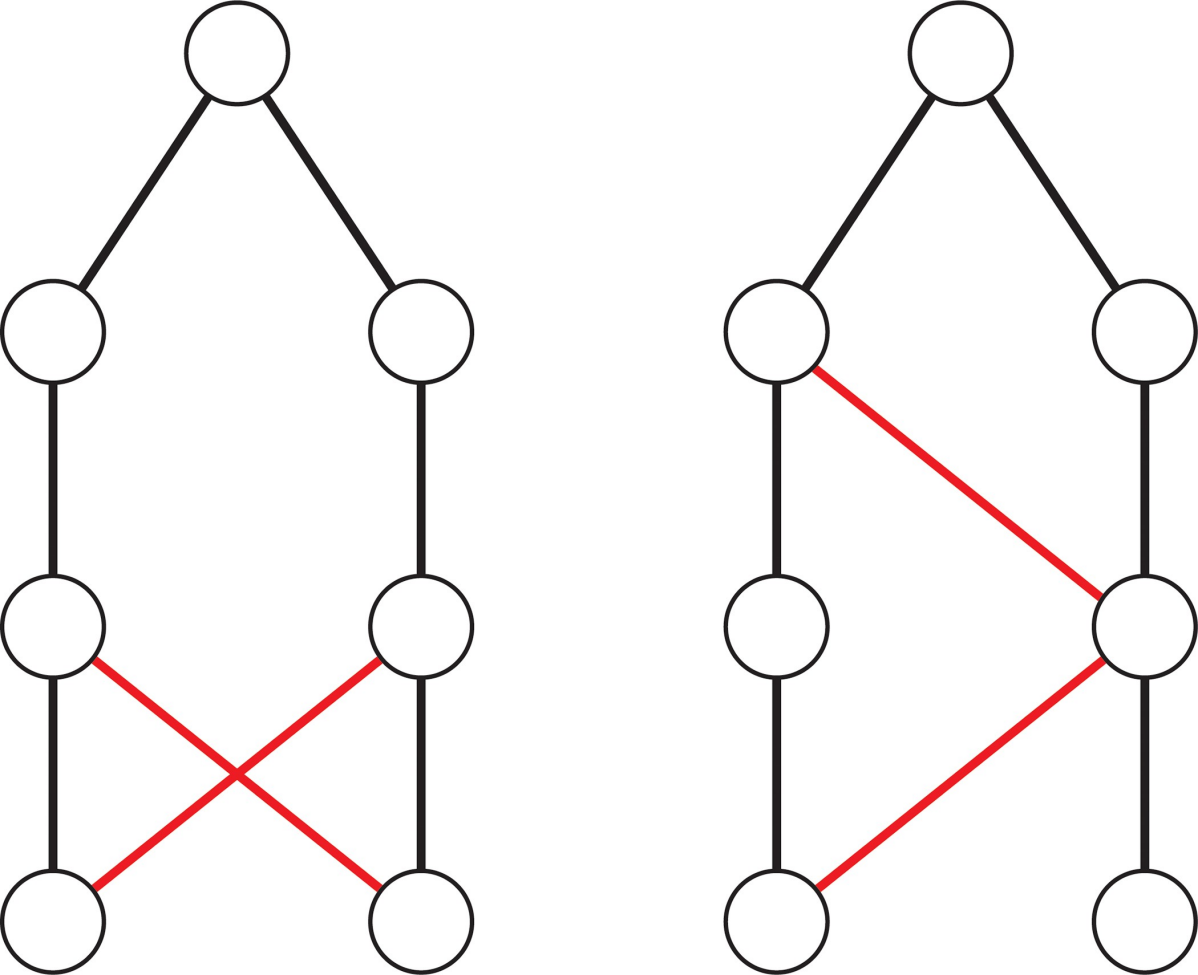
Supervisor / examiner relationships delayed by one generation. Black lines denote supervisor relationships. Red lines denote examiner relationships.

If reciprocity is delayed by a generation then relationships appear less incestuous in an academic context: we could imagine either students of two students of one supervisor examining each other (‘first cousin once removed’ examining) or the return occurring in a later generation, see Fig 4.

We should stress that these sort of loops may be emergent properties of localised strategic decision making rather than being deliberately crafted. We are not suggesting that examiners are being chosen because of being intellectual uncles or aunts of the student concerned. In some cases, however, these are the people best placed to act as examiner and unintended consequences of such assessments is that cycles are created. In our data, we found 48 examples of simple closed loops and 36 examples of Incestuous closed loops.

### 3. Analysis

There are some simple statistics to collect to get a sense of the graph before we look at network statistics. A crude measure of prestige could be associated with the number of doctoral students that a researcher has supervised. Being successful, prominent or of high status in a discipline is likely to lead to an individual attracting research students, and to be being asked to serve as examiner for many others. On the face of it there is potentially a gradient from someone with a newly minted doctorate who has neither themselves been an examiner nor supervised any students to a senior figure who at retirement has supervised and examined many students. To explore this intuition, we took the topmost individuals in the lists of most supervisees (Table 2) and most examinees and collated the awards and other tokens of prestige they had received.

**Table 2:** Top supervisors in the field of primatology by number of supervisees. Only those with more than 10 students are shown.

(node_id) Name	Number supervised
(Merge_02898) Dunbar, Robin I. M.	32
(AU_16114) Sussman, Robert Wald	29
(AU_16017) Jolly, Clifford J.	27
(Merge_07191) Martin, Robert D.	25
(AU_16069) Washburn, Sherwood Larned	23
(AU_16040) Lee, Phyllis Chadwick	21
(TempID_211) Hinde, Robert	21
(AU_16019) Wrangham, Richard W.	20
(AU_16375) Dolhinow, Phyllis Jay	20
(Merge_09577) Rodman, Peter S.	20
(AU_16024) Richard, Alison Fettes	19
(AU_16048) McGrew, William C.	17
(AU_16091) Tuttle, Howard Russell	17
(AU_16105) Snowdon, Thomas Charles	17
(AU_16085) Simonds, Paul Emery	16
(TempID_537) Whiten, Andrew	16
(AU_13219) Wright, Patricia Chapple	15
(AU_16492) Nishida, Toshisada	15
(AU_16562) Van Schaik, Carel P.	15
(AU_16088) Devore, Irven	14
(AU_16096) Poirier, Eugene Frank	14
(AU_16190) Cords, Ann Marina	14
(AU_16097) Bramblett, Claud Allen	13
(Merge_04834) Holekamp, Kay	13
(AU_13024) Glander, Kenneth Earl	12
(AU_16018) Oates, John F.	12
(AU_13047) Godfrey, Laurie Ann Rohde	11
(AU_13151) Harrison, Terry	11
(AU_13175) Watts, David Peter	11
(AU_16106) Pilbeam, Roger David	11
(AU_16118) Delson, Eric	11
(AU_16125) Kay, Richard Frederick	11
(AU_16137) Fedigan, Linda Marie	11
(Merge_01276) Buikstra, Jani E.	11

To motivate the use of a network-statistic approach to gain insights we look at a slightly more fine-grained statistic that is natural to derive from the course-grained statistics in Table 2. The number of examinations in which an examiner has participated, in Table 3, can be misleading since in the committee and jury systems many supervisors are members of their own students’ doctoral committees/juries. We have attempted to allow for this by counting only examinations of students that are supervised by others and by weighting the figures to reflect the difference between internal and external membership of juries and committees. For the UK examination system where the norm is only two examiners, we have included only the external examiners and have not recorded internal examining.

**Table 3:** Ranking of primatologists by examining engagements.

(node_id*) Name	Number examined	Number own students examined	Number of others examined	Weighted no. examinations (External + 0.5 internal)
(AU_16114) Sussman, Robert W.	41	15	26	33.5
(AU_16017) Jolly, Clifford J.	56	36	20	38
(Merge_09577) Rodman, Peter S.	22	6	16	19
(AU_16375) Dolhinow, Phyllis J.	18	3	15	16.5
(AU_13219) Wright, Patricia C.	35	21	14	24.5
(AU_16096) Poirier, Eugene F.	19	7	12	15.5
(AU_16019) Wrangham, Richard W.	35	24	11	23
(AU_16097) Bramblett, Claud A.	17	6	11	14
(AU_16148) Fleagle, John G.	45	35	10	27.5
(AU_16091) Tuttle, Howard R.	19	9	10	14.5
(Merge_05008) Howell, Francis C.	18	8	10	14
(AU_16069) Washburn, Sherwood L.	16	6	10	13
(Merge_04834) Holekamp, Kay	14	4	10	12

*node_id refers to the id column of the DATA_names.txt file in [13].

To reflect the relative perceived merit of internal versus external examination we introduce an arbitrary relative weighting to make a point. In our weighting of committee /jury members external members are weighted 1, internal at 0.5. Where affiliation is not recorded, as sometimes occurs in the data (in our dataset, 0.4% of thesis authors and 6% of authors and examiners overall lacked affiliation information), we assume they are internal so weight them by 0.5.

Note that if we look purely at the data in Table 3, this is a difficult half-way house between a very pure high-level statistic and a measure that attempts to give us context. If you look in isolation at the number of examinations then you may think that Clifford Jolly is the most highly esteemed academic. However, number of external examinations might be a better proxy and so Jolly, Wrangham and Wright all do well on this measure. However, using our arbitrary weighting Sussman scores extremely highly. The arbitrariness of the weighting that we’ve introduced makes sense instinctively but skews the numbers. So, our attempt to account for effects that we know about by adding in an arbitrary variable only makes things worse.

Indeed, the problem is significantly more complex when you try to add further context. At the centre of the problem is the lack of context built into the statistics that we have available. In what communities do these individuals sit: are they at large institutions? Do they have funding to travel due to generous research funding environments? Are they based in one of the geographical regions in which there are many universities (for example New York, Boston, London or Melbourne) and so invitations to participate in external evaluations may be more plentiful since travel costs can be kept low? No high-level analysis is ever going to capture that level of nuance and for a group of thousands of people it is impossible to capture enough variables to truly represent this. However, we argue that the structure of the network that we’ve build should encode some of this “landscape” information and hence network statistics should provide more contextualised norms. In some sense, the weighting that we codified manually should already be encoded in the network structure and should emerge through the ranking.

Although there was a high degree of correlation between many of the network and non-network measures there were some individuals identified by network measures who would have been missed by the simple counting exercise and which, perhaps, more closely represent an “insider’s” intuitions e.g. that Washburn was an important figure, founding a lineage. Significantly, though Washburn is identified as important by all measures so is Howard Russell Tuttle.

Given that network statistics have a slightly different flavour to mere counting it is important to understand how closeness centrality and betweenness centrality work. In his recent work, *the Square and the Tower*, Naill Ferguson gives an excellent account of network statistics [22], while more technical treatments may be found in [23,24]. Closeness centrality is calculated as the sum of the length of the shortest paths between the node and all other nodes in the graph. It is important because it gives a sense of the directness connection of any node to any other node. In academic terms, those with high closeness centrality tend to be established academics with developed social networks arising from a rich collaborative career and significant conference attendance or positioning in a connected academic institution. In some sense, closeness centrality can be a good measure of “establishment” or “fame”. The *betweenness centrality* of each node is defined in terms of the shortest paths between pairs of points in the graph. The node with the highest betweenness centrality is the node through which the highest number of shortest paths pass. In a sense, this is the node which is most likely to be “on the way” from any random choice of node to any other random choice of node. Betweenness gives us a measure of how strategically a node is connected. For example, if a graph may be decomposed into two equal internally-connected but overall disconnected graphs and one new node is introduced with a single connection to each of these two graphs, then it is instantly a candidate to be the node with the highest betweenness since all paths must pass through that node which wish to travel from one graph to the other. In academic terms, we find people with high betweenness in many scenarios but particularly in the early stages of emergence of interdisciplinary communities. Those academics who connect two disparate communities have high betweenness in co-authorship networks as there are few links between those communities. Betweenness can be a good measure of innovation or a signal for certain types of collaboration. We have calculated these two values for all academics in our network and show the most highly ranked in Table 4.

**Table 4:** Lists the top ten individuals for five different network measures (and those supervising/examining >10 theses). We then removed those identified by only one measure then look at the individuals who are prominent by several different measures. Closeness Centrality and Betweenness centrality have the standard definitions. Output domain (degree) of an individual is the number of all people to whom each actor is connected. Output proximity prestige of an individual is the proportion of all other vertices in their output domain divided by the average distance from this individual to the other people in their output domain (that is, normalized output domain / average distance). The citation weight of an individual (using the Search Path Count algorithm) is the normalised sum of direct descendants.

Closeness centrality		Betweenness centrality					Output domain					Output proximity prestige		Citation weight		Supervising > 10 (abridged; number in parenthesis denotes ranking in full list)		Examining > 10	
AU_16069	0.12	AU_16069			0.06		TempID_219			747		AU_16069	0.03	TempID_219	0.06	AU_16114 (2)	29	AU_16114	26
AU_16148	0.12	AU_16148			0.03		AU_16069			740		TempID_219	0.02	AU_16069	0.06	AU_16017 (3)	27	AU_16017	20
AU_13024	0.12	Merge_09577			0.03		Merge_11021			325		AU_16091	0.01	Merge_11021	0.03	AU_16069 (5)	23	Merge_09577	16
AU_16091	0.12	AU_16005			0.03		TempID_256			323		AU_16075	0.01	AU_16068	0.03	AU_16019 (8)	20	AU_16375	15
AU_16088	0.12	AU_16091			0		AU_16068			322		AU_16088	0.01	TempID_256	0.03	AU_16375 (9)	20	AU_13219	14
AU_16114	0.11	AU_16088			0.02		AU_16091			317		AU_16017	0.01	AU_16091	0.03	Merge_09577 (10)	20	AU_16096	12
Merge_09577	0.11	AU_16017			0.02		TempID_241			307		Merge_11021	0.01	AU_16075	0.02	AU_16024 (11)	19	AU_16019	11
AU_13242	0.11	AU_13024			0.02		AU_16075			306		AU_16068	0.01	TempID_241	0.02	AU_16091 (14)	17	AU_16097	11
AU_16017	0.11	AU_13197			0.02		AU_16088			268		AU_13024	0.01	AU_16088	0.02	AU_13219 (16)	16	AU_16148	10
AU_1084	0.11	AU_16024			0.02		Merge_03510			188		TempID_241	0.01	Merge_03510	0.01	AU_16088 (21 = )	14	AU_16091	10
																AU_16096 (21 = )	14	Merge_05008	10
																Merge_04834 (24)	13	AU_16069	10
																AU_13024 (25)	12	Merge_04834	10
																AU_16148 (28 = )	11		
**Occurrences:**		**7**	**6**	**5**		**4**		**3**	**2**		**1**								

**Table 5:** Ranking of academics, with node ID's associated with names, from Table 4 with the same colour coding. We flag were an academic has only appeared in centrality measures.

Node ID	Name	Appears in # measures	Appears only in centrality measures
AU_16069	Washburn, Sherwood	7	
AU_16091	Tuttle, Russell	7	
AU_16088	Devore, Irven	6	
AU_16017	Jolly, Clifford	5	
AU_13024	Glander, Kenneth	4	
AU_16148	Fleagle, John	4	
Merge_09577	Rodman, Peter	4	
AU_16068	McGrew, William	3	x
AU_16075	Simon, Elwyn	3	x
AU_16114	Sussman, Robert	3	
Merge_11021	Srebnik, Herbert	3	x
TempID_219	Hooton, Earnest	3	x
TempID_241	Jepsen, Lowell	3	x
AU_13219	Wright, Patricia	2	
AU_16109	Wrangham, Richard	2	
AU_16024	Richard, Alison	2	
AU_16096	Poirier, Eugene	2	
AU_16097	Bramblett, Claud	2	
AU_16375	Dolhinow, Phyllis	2	
Merge_03510	Dunbar, Robin	2	
Merge_04834	Helobamp, Kay	2	
TempID_256	Keith, Arthur	2	x

Table 5 combines a number of different network statistics that we deem to be important in assessing the importance of an individual to the social community. Twenty-two individuals are identified by at least two of the different measures considered and we discuss these below.

We note that the following six individuals are identified by centrality measures but do not appear in the lists of examinations or supervisors: Elwyn Simons, Herbert Srebnik, Earnest Hooton, Lowell Jepson, Arthur Keith, William McGrew. Among these is Earnest Hooton (d 1954), Washburn’s supervisor. One interpretation of Hooton’s position is that his centrality is an effect of that of his student, he has, as it were, acquired some of the prestige of his illustrious student. Arthur Keith (d 1955) is the other older figure in our list, and a surprising one: he is usually regarded as a human paleontologist although he did study primate skeletons. The two measures in which he scores well are both those which, in effect, reflect the prominence/success of descendants (‘output domain’ and ‘citation weights’. For the latter measure, readers should remember that these are not actual publication citations, rather we are using a measure first developed to analyse networks of citation patterns).

Conversely, we also note that the list includes Kay Holekamp, a senior zoologist who is not a primatologist by specialization. She enters our list as a prominent supervisor and examiner of primatologists.

Herbert Srebnik, Professor of Anatomy at Berkeley is of note since he is not a prolific examiner or supervisor nor has he been widely recognised by the award of medals etc. However, he has clearly played an important role as reflected in his pivotal position in the networks of supervision and examining.

### 4. Ranking theory and prestige

In this section, we extend the ideas of the previous section to explore our genealogy network in the context of ranking theory. Here we wish to improve our “computation intuition” by adding features to our graph that allow us to not only assess the relative prestige of individuals but see how prestige has a dynamics associated with evolution of the network.

A reader’s first impression of someone’s curriculum vitae is influenced by the university that they attended, their doctoral advisor, examiners and a number of other factors. If we consider each participant in the network to be a node with a “score” defined by some objective assessment of their vitae and links between nodes provided by an academic hierarchy such as PhD mentorship or PhD examination (or a combination of these two) then we define a “system of prestige”. If we wanted to understand the relative prestige of one person with respect to another in the network it is important to understand the context surrounding each person and one can use network analytic approaches to quantify the relative prestige of any two people on a network that is “strongly connected”. (We won’t define technical terminology here since it is not needed to follow our arguments, but instead refer the reader to [5,25].) Hence, when we talk about prestige evolving with the evolution of the network. The implication is clear: Each connection shared between people allows a little “glory” to be reflected in each direction. It is important to understand that when we talk about flow, we are not talking about a dynamical quantity on the network. Rather we are describing the rebalancing of the prestige scores at each point in the network when new information is added to the network. New information in this context can come from three types of event: i) the addition of a new edge–where a new qualification event takes place and links nodes that already exist in the network; ii) the addition of a new node as a result of activity i); and iii) the exogenous addition of new prestige–for example, a new prize or honour conferred a participant in the network. Assessing this network to calculate the relative prestige of the participants can be classed as a ranking problem. This is a well-studied problem that has been seen in many contexts from Google’s PageRank algorithm to modern content recommendation engines and even College Football rankings [5, 25].

Typically, network approaches require a way to assign scores to each node in the graph to create a “preference matrix”. This preference matrix is then used to create a ranking that takes into account not only the strength of each individual node individually, but the strength of the nodes that have relationships with that node, as well as second, third, and eventually *n*th level relationships. The work of Keener [5] shows that the construction of the preference matrix in a correctly-normalised manner preserves the probabilistic interpretation of the network and means that network statistics such as betweenness centrality and closeness centrality continue to be valid concepts.

While the network that we’ve studied is directed in time, if prestige is defined in terms of network statistics then as the network develops prestige (or ranking) of every node changes as new connections and new prestige events (awards, fellowships and so on are conferred). Hence, prestige can be interpreted to flow both forward and also backward in generations. For example, from our previous discussion, it is clear that having a PhD advisor or examiner who is the recipient of a Nobel Prize can be good for the student or examinee. However, the advisor to a student who goes on to win a Nobel Prize also benefits from an enhanced reputation. Hence, a network of academic relationships with different prestige factors associated with different individuals gives rise to a constantly rebalancing and shifting system of prestige.

An excellent example of PhD advisor who never won a Nobel Prize himself is the German physicist Arnold Sommerfeld who, as a PhD advisor produced Werner Heisenberg, Wolfgang Pauli, Peter Debye and Hans Bethe, all of whom won Nobel Prizes in Physics or Chemistry. These were not the only famous names that he supervised (although not necessarily at doctoral level) he worked with Linus Pauling and in more mathematical matters with Ludwig Hopf, Rudolf Peierls and Wilhelm Lenz, all of whom made significant innovations in their chosen fields that are in use regularly by modern-day practitioners. Sommerfeld himself is well known to field theorists for the Bohr-Sommerfeld quantisation condition and a number of other techniques. He won the Max-Planck medal, the Lorentz Medal and become a Fellow of the Royal Society–all significant measures of prestige in their own right but not perhaps with the lustre of a Nobel Prize. Yet Sommerfeld’s reputation is certainly enhanced by having been associated with some of the great names in quantum physics and having supervised more Nobel Prizes winners than anyone else (with the possibly exception of the English physicist JJ Thompson). Conversely, Richard Feynman dominated theoretical physics in the 1950s and 1960s winning a Nobel Prize for his contribution and is occasionally described as the “smartest man alive” in that period. Yet, none of his students won a Nobel Prize.

In formulating our ranking we need to associate scores with each researcher. In the ranking created by Keener [5] for football teams, this score could be derived from the number of goals achieved by each side. Hence, in the Keener case, each game or interaction relates a pair of teams and these scores are summed over the season. In our academic case, the picture is more static. A scoring method needs to be defined such that when an honour is conferred on an individual, the node weight is adjusted to take this into account. This is where a lot of subjectivity is involved in any ranking scheme. We see precisely this approach applied to university rankings, where different data and different weighting schemes lead to different outcomes. Here we are attempting to make personal rankings more reflective of the context of the person (i.e. taking account of the interactions that we can derive from the relationships that people have in the form of qualification events, which are the analogue in this model of the football game interactions). The effect of using the approach in [5] to rank the nodes in the network is that the final ranking is not simply the sum of the weights associated with the awards that each person has but rather it takes into account the network effects of being located in the network in proximity to an award winner. This makes sense since, if a prize winner chooses a doctoral student, then they are likely to have had a good field to choose from and the chances that the student may be good is higher. There is also a reinforcing effect where that student, being supervised by a prize winner, is more likely to be afforded opportunities than other students. The network ranking approach associated with [5] models this type of reinforcement in a natural way. The ranking scheme is also quite robust, which is to say that the model deals with partial information quite well. Typically, if scores can be assigned to the square root of the number of nodes in the network, a workable ranking can still be derived with some confidence.

In order to specify the elements of the preference matrix described above, we need to suggest a scoring methodology. As noted earlier, each node corresponds to either a supervisor, an examiner or a degree candidate. The weight that we assign to each node in the network should describe their achievements or the prestige that has been conferred on the individual so far. Hence, our sketch formula for the weight of each node/researcher might be:

Weight=a∑NobelPrizes+b∑ElectedFellowships+c∑Awards+d∑HonoraryDegrees+e∑Editorialboardmemberships+f∑Grantcommitteememberships+⋯

where the sum in each case is over all the awards won in each category by the person in their career so far, and each coefficient (a,b,c,…) represents a universal weight associated the type of award in the sum. Ideally, these universal weight factors are determined by surveying a large population of practitioners so that the relative strength of the awards are well understood. In practice, and in many cases where such methods are used in university rankings, these weight factors are either arbitrarily decided, hidden from those being ranked, or both. We normalise these weights (a, b, c, …) by summing across the nodes in the graph and dividing out. We note that modifying the weight function above by removing the co-efficients and summing reduces to the sum of awards given. Hence, the sum of the weights is a quantity that is something like the total “prestige” given. These concepts are analogous to particle number and energy preservation in a closed physical system. The weight function can be directly considered to be the “bare” prestige associated with an individual, however, it does not directly represent the network effect of being connected to other nodes in the graph. The output of the ranking approach described here would be a “ranking vector” that gives a relative score to each node in the graph. This approach is entirely analogous to Google’s PageRank algorithm. In this context, we define *academic prestige* as PageRank of academics in a connected academic genealogy in which weights are determined by a weighting function provided by the honours that each academic in the network has received. The precise formulation of the weighting function is defined by the user.

There are many more element types than we have listed in this short example that may be important to consider, for example these may include many well-established measures of prestige such as honorary chairs, named chairs, prestigious committee memberships, government advisory positions, through to more modern prestige measures such as having a Wikipedia page. In each case, there are subtle interplays between different elements, which need to be understood to calculate an overall score. For instance, the example above contains no interaction terms between awards. An example where it may be necessary to consider such interactions is the winning of a Fellowship of the National Academy of Science in the US. When an American researcher receives the Nobel Prize in their field then they will almost without question by invited to become a member of the National Academy of Science. Is it appropriate that they should get a “booster” to their prestige score by this or is it entirely right and the halo effect of a Nobel Prize should propagate unseen in our equation?

The prizes and elected fellowships as well as other awards and measures of prestige that we suggest above have a constancy or a quality that we contend is approximately constant in time. Hence, we borrow the concept of “standard candle” from astronomy. In astronomy, standard candles are types of celestial object that burn with a luminosity that is well understood and hence they can be used to calculate their distance. We posit, for an example, that a Nobel Prize always takes about the same level of academic achievement and command of a field relative to others and that, as a result of winning such a prize, the prestige of the recipient will manifest in approximately the same way at any point in time. Hence, by using the prestige measure that we suggest above, we can compare the relative prestige of researchers across generations.

Even for a small, tight-knit community such as primatology, not everyone is connected in a single graph. There are colleagues who work on distinct topics and who form disconnected sub-groups. In Table 1, we summarize the sizes of the largest connected components of the graphs that we have worked with in this paper. The large connected components contain 65–70% of the nodes in the data. The network ranking approach that we have suggested here does not have the capacity to create a full relative ranking of all components if they are not part of the same connected piece. In exploring the problem further, we would need to work with the largest connected component for the chosen ranking method to be applicable.

Collating information about honours proved difficult. This was partly due to the difficulties of handling heterogeneous data. In particular, we encountered several questions as we looked deeper into this line of exploration: Which awards do we decide to be significant? Does the significance of awards change with time? Are records of award that are publicly available sufficiently complete? Other issues are also raised: How does one assess the relative merit of being elected to membership of Sigma XI or the British Academy over having discovered a new species? On the basis that success breeds success or that consistent success is reflected over a career by the accrual of awards and prizes we made a crude count of identifiable honours.

When counting the measures of prestige awarded to a key group of 22 individuals identified by the network analysis in Tables 4 and 5 there is no clear correlation. Only Russell Tuttle, scores highly on both, and there is no statistically significant correlation (see Table 6). Table 6 includes counts of many honours including: Fellowship American Association for the Advancement of Science, Fellowship of the American Academy of Arts and Sciences, the Alban-Heiser Award for Conservation of the Houston Zoological Society (for a full list please see ancillary material).

**Table 6:** List of academics showing correlation between number of real-world honours received (Honour Count) and significant position in academic network analysis (Top 20 nodes by network centrality measure).

Name	Honour Count	Network Measures Count
Dolhinow, Phyllis	1	2
Poirier, Eugene	1	2
Rodman, Peter	1	4
Srebnik, Herbert	1	3
Bramblett, Claud	2	2
Sussman, Robert	2	3
Jolly, Clifford	3	5
Holekamp, Kay	5	2
Jepsen, Lowell	5	3
Keith, Arthur	7	2
Washburn, Sherwood	7	7
Glander, Kenneth	9	4
Devore, Irven	13	6
Dunbar, Robin	16	2
Hooton, Earnest	16	3
Simons, Elwyn	16	3
Fleagle, John	19	4
Tuttle, Russell	21	7
Wrangham, Richard	25	2
Richard, Alison	26	2
McGrew, William	27	3
Wright, Patricia	37	2

### 5. Discussion

What this exercise has shown is that combining supervisory and examiners data is powerful. It enables us to identify individuals who are judged on the basis of their publications and other activity (including doctoral supervision and examining) to be of merit. In the UK context this means that metrics (or ‘indicators’ see Ref. 22) based on supervisory and external examination activity might be of interest to HEFCE in their regular Research Evaluation Framework exercises.

In the long term we hope that this pilot case will encourage the keepers of the metadata, the directors of DART (Digital Access to Research Theses), Proquest theses database and so on, to include examination data with agreed metadata standards so that this sort of analysis can be undertaken across all academic disciplines and evaluated across time.

There are many potential directions for new research projects based on the ideas in this paper and we only indicate a few here. It is interesting to solidify the model that we have sketched by surveying broadly to find appropriate weightings for each of the prestige factors in the weight equation. Extending the model so that field dependence is taken into account with some appropriate normalisation is important.

A further fruitful area for future enquiry is the study of the relative weights for supervisors and examiners in the genealogy. Can a generalised rule be developed for the relative weighting or should the weighting be dependent on the relative perceived strengths of the examiner and supervisor nodes?

It may also be interesting to study the “horizon of last influence”. That is, the longest time back that any “significant” change can be made to the perceived prestige of a colleague–a clear definition of “significance” needs to be defined for this to be possible. We postulate that this is field independent since the effect of a supervisor’s supervisor on a student may be minimal and a 3^rd^ degree relationship is almost certainly even less. However, particular fields may have especially close networks, particularly short generation times or the effects of major prizes such as the Nobel prize may have a disproportionate effect on the structure of those fields in which they exist.

Another direction is considering the use of community detection and pairing this with ranking to look at the distribution of awards on a naturally emergent subject categorisation. It is clear that some research areas profit from more awards than others, hence, combining the work of Evans and Lambiotte [26,27], and Hutchins et al. [28, 29] suggests interesting possibilities for clustering and local rankings within academic “families”.

To summarise, we have demonstrated that intellectual genealogies can be enhanced by going beyond the supervisor–student relationship, and that to properly understand the lineages we need to consider bilateral kinship structures. The important figures in the networks are those who are recognized as high status through the award of prizes, medals and prestigious fellowships. These prizes form the basis of our perception of who is most influential in academic networks. However, prestige/esteem is a more complicated quantity and proximity to those who hold prestigious awards in the academic genealogy can be a signal for unseen influence or future success depending on career stage.
